# Murine Aseptic Surgical Model of Femoral Atrophic Nonunion

**DOI:** 10.1016/j.mex.2020.100898

**Published:** 2020-04-21

**Authors:** Ryan R Kelly, Mary Ann McCrackin, Dayvia L Russell, Lee R Leddy, James J Cray, Amanda C LaRue

**Affiliations:** a Research Services, Ralph H. Johnson VA Medical Center; b Department of Population Health, College of Veterinary Medicine, University of Georgia, Athens, GA; c Department of Orthopedics, The Medical University of South Carolina, Charleston, SC; d Division of Anatomy, The Ohio State University, Columbus, OH; e Department of Pathology and Laboratory Medicine, The Medical University of South Carolina, Charleston, SC

**Keywords:** Orthopedics, Surgery, Mouse, Nonunion, Fracture, Asepsis, Animal model

## Abstract

Although bone repair is typically an efficient process, an inadequate healing response can occur, with approximately 5-20% of fractures developing nonunion. Even with improved healing strategies and external fixation devices, overall rate of nonunion has not been significantly reduced, particularly for atrophic nonunion. Atrophic nonunion is characterized by sparse or no callus formation and is difficult to treat clinically, resulting in long-term pain and functional limitation. Reliable preclinical models are needed to study the pathophysiology of atrophic nonunion to create better treatment options. The MouseNail kit (RISystem, Landquart, Switzerland) provides a highly standardized approach in which stabilized segmental bone defects are achieved through interlocked intramedullary nailing. However, reliably performing this surgery is technically challenging, particularly while maintaining strict asepsis. Skilled and aseptic surgical execution is important and necessary because it ensures optimal animal welfare and reproducibility. Therefore, the aim of this paper is to describe:

• Novel modifications to the MouseNail kit that allow for: 1) a completely aseptic surgical environment, including description of a hanging limb orthopedic aseptic preparation and 2) a reduction in fracture gap size necessary for induction of atrophic nonunion.
• Pre- to post-operative recommendations to facilitate successful performance of murine orthopedic survival surgery.

## Graphical Abstract

**Figure:**
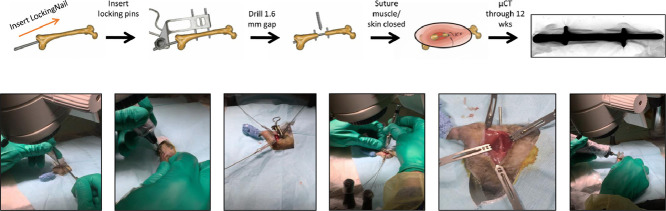

**Table:** Specifications Table

Subject Area:	Medicine and Dentistry
More specific subject area:	Mouse atrophic nonunion
Method name:	Aseptic application to existing atrophic nonunion model
Name and reference of original method:	Garcia P, Herwerth S, Matthys R, Holstein JH, Histing T, Menger MD, Pohlemann T. The LockingMouseNail–a new implant for standardized stable osteosynthesis in mice. The Journal of Surgical Research. 2011. 169: 220–226. PMID: 20371084.
Resource availability:	https://risystem.com

## Method details

It is estimated that 100,000 fractures in the United States will progress to nonunion each year [1]. Even with improved healing strategies and external fixation devices, the overall rate of fracture complications has not been significantly reduced. Atrophic nonunion is defined as a nonunion with sparse or no evidence of callus formation. This subtype is particularly challenging to treat because, rather than a mechanical problem, it is viewed as a biological problem. While the pathophysiological mechanisms are largely unknown, incidence of atrophic nonunion is multifactorial. It may be avascular and require a bone graft in addition to fixation. Atrophic nonunion is associated with a prolonged treatment schedule, resulting in pain, functional, and psychological disability. Creation of segmental defect in preclinical models is likely the best approach for mimicking the clinical situation of impaired healing, as it can be readily standardized and allows for analysis of the effects of scaffolding, cell transplantation, and implantation of osteogenic factors at the defect site. Pre-clinical models for nonunion have been described in rodents, rabbits, and sheep. In regard to mouse models of atrophic nonunion, the MouseNail kit (RISystem, Landquart, Switzerland) provides a highly standardized approach in which stabilized segmental defects are achieved through interlocked intramedullary nailing, resulting in a high degree of axial and rotational stability [2]. This approach was shown to reliably generate atrophic nonunion in CD-1 mice [3]. Studies have shown that reliable generation of fractures is dependent on mouse strain used, with C57BL/6 demonstrating a rapid healing rate [4]. Given the widespread use of the C57BL/6 strain, standardized surgical methods for generation of atrophic nonunion in this strain are needed.

In this method, we describe how we have generated atrophic nonunion in a cohort of C57BL/6 mice using aseptic methods in conjunction with novel modifications to the MouseNail system. Atrophic nonunion had previously been achieved with a fracture gap size of 2.0 mm using the MouseNail kit [2]. However, for development of translational orthopedic therapies, it is vital to generate atrophic nonunion with a critical-sized defect that has the potential to be healed with intervention. Our findings suggest that atrophic nonunion can be reliably achieved with a critical-sized defect of 1.6 mm when both locking pins are properly installed. Alternatively, by installing one locking pin, delayed union can be created. Our goal was to create a surgical protocol that was repeatable, precise, and utilized aseptic techniques to the same standard used in large animal surgical models. Previous publications describing implantation of the MouseNail do not address the application of standard of care asepsis necessary to meet research regulatory requirements in the United States. In fact, a published video, although providing a clear visual guide for performing the technical aspects of the surgery, demonstrates placement of the MouseNail using a cadaver mouse without shaving of hair on the subject limb or aseptic preparation [5]. Our intention was to maintain the best possible environment to maximize animal welfare, which, in turn, positively affects data quality and minimizes complications pre- and post-operatively. The novel modifications and aseptic conditions developed in our model can increase reproducibility of this technically challenging surgery in order to study the basic mechanisms of atrophic nonunion fracture and utilize this knowledge to examine the efficacy of clinically relevant therapeutic strategies. We have also used this surgical protocol as a general basis for our pre- through post-operative recommendations and considerations provided under Additional Information for performing murine orthopedic survival surgeries.

### Atrophic nonunion femoral fracture using aseptic technique

Below, we provide our complete surgical protocol for performing aseptic technique using the MouseNail kit to create femoral atrophic nonunion fracture. This study was conducted using 12-14-week-old male C57BL/6 mice. All procedures were performed under protocols approved by the Institutional Animal Care and Use Committee of the Ralph H. Johnson Veterans Affairs Medical Center Research Service, a Public Health Service-assured and AAALAC International-accredited animal care and use program.

**Fig. 1:**
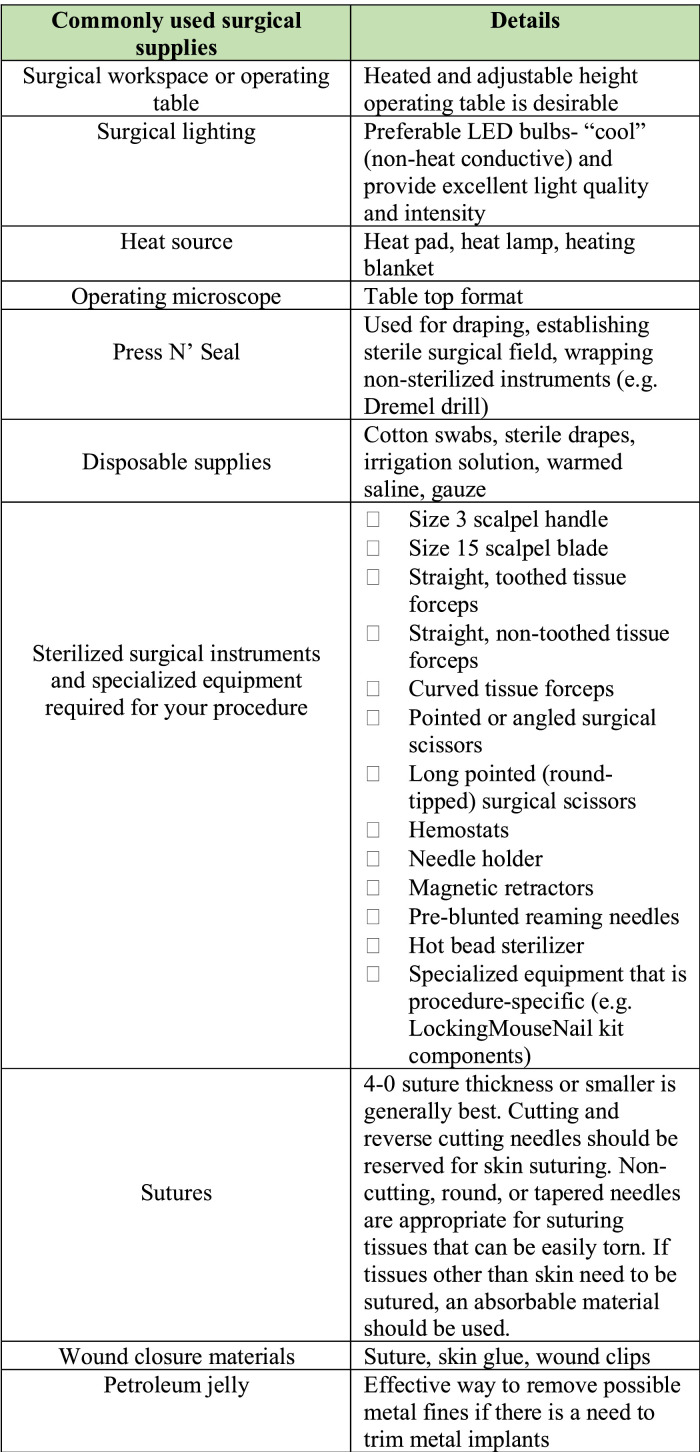
Common supplies required for surgical operation. Surgical instruments and supplies are outlined with detail.
• Sterilize surgical instruments (we use steam sterilization via autoclave).
• Set up anesthesia equipment (e.g., place tubes and nose cones in appropriate areas; weigh charcoal canisters if passive scavenging is used).
• Prepare mouse recovery cages (sterile compressed cotton bedding; Shepherd's™ ALPHA-dri^Ⓡ^) and rodent health monitoring cards.

Initial Set-up:

• Ensure you have necessary instruments and supplies for surgery (Fig. 1).

**Fig. 2:**
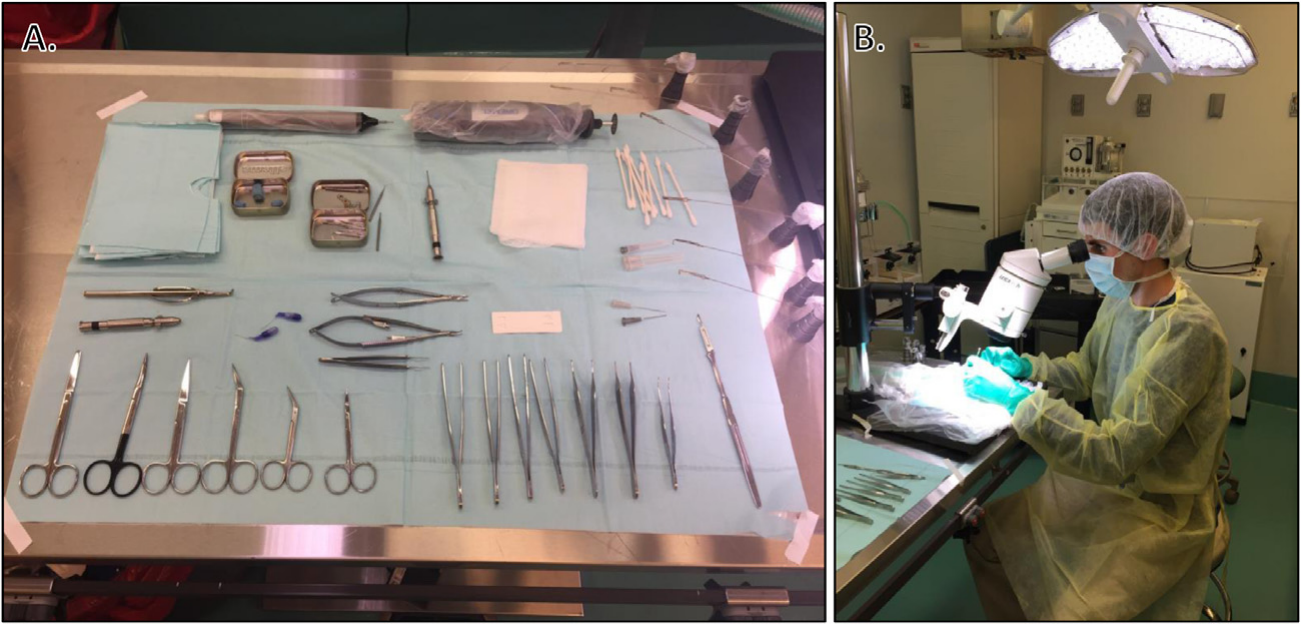
Instrument preparation for surgery. A) Surgical instruments are laid out on sterile drape beside operating microscope to allow surgeon easy access during surgery. Note that surgical instruments can only be arranged in the organized manner shown here after donning surgical gloves (i.e., immediately before starting surgery). During initial set-up, open the autoclaved instrument packs, ensuring not to touch instruments or inside surfaces of packaging, and let instruments fall onto sterile drape. B) Surgeon is performing orthopedic procedure through operating microscope.
• Wrap in Press'n Seal^Ⓡ^ any instruments or supplies that could not be autoclaved, taking care to wrap while not touching area of instruments that will be handled during surgery.
• Prepare surgical microscope and lighting.
• On top of surgical microscope stage, lay down layer of Press'n Seal^Ⓡ^, place warming pad on top (we use WARM GEL™ Instant Heat Pack, PRISM Technologies, Norcross, GA), and then add additional layer of Press'n Seal^Ⓡ^ on top of heating pad.
  ○ Note: choice of warming equipment must be carefully considered to limit risk of thermal burn to mouse

Preparation of Surgical Field and Surgeon:

• Don personal protective equipment, including shoe covers, disposable gown, nonsterile gloves, face mask, and bouffant cap.
• Lay sterile drape where sterile instruments will be placed and tape down the corners using waterproof first aid (take care to only touch outside edges of drape to avoid contamination). Alternatively, the entire support area can be covered with Press'n Seal^Ⓡ^ (Glad^Ⓡ^, Oakland, CA) and ends wrapped under table edges. Press'n Seal^Ⓡ^ is a sterile wrapping that can be used as a cost-effective alternative to sterile drapes in rodent surgery. It is particularly useful for wrapping instruments that cannot be autoclaved [6].
• Open autoclaved instrument packs, ensuring not to touch instruments or inside surfaces of packaging, and let instruments fall onto sterile drape (Fig. 2).

**Fig. 3:**
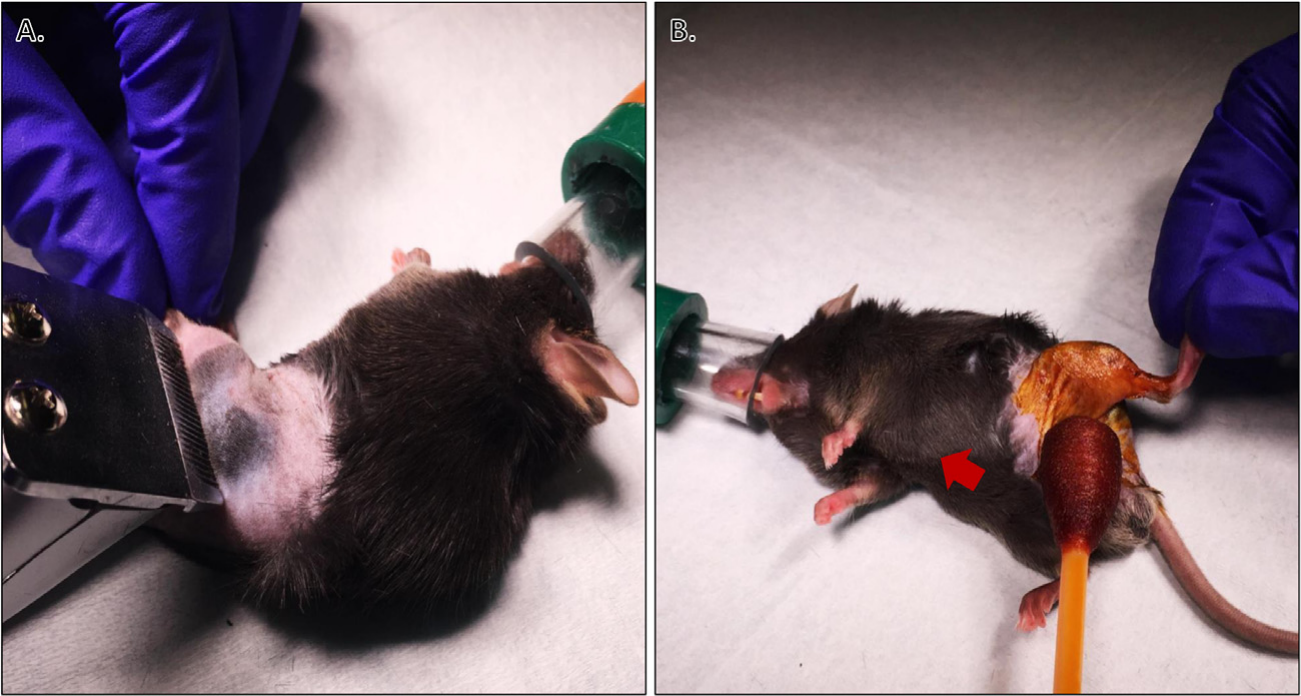
Preparation of surgical site. A) Shaving of surgical site to remove fur. B) Application of Betadine^Ⓡ^ as part of aseptic preparation of the surgical site. Red arrow indicates xyphoid process for reference.
• Use alcohol wipe to remove as much shaved fur as possible.

Mouse Incision Site Preparation:

• Approximately 30-45 min prior to start of surgery, perform a subcutaneous injection of buprenorphine (0.1 mg/kg).
• At 10-15 min before start of surgery, place mouse inside anesthesia chamber.
• Isoflurane should be turned to ~3% for induction and adjusted down to ~2% for maintenance (percentage will vary among individual mice and depend on pre-operative medications used).
• Allow mouse to stay in chamber until breathing is steady but slow.
• Perform toe pinch to verify loss of withdrawal reflex, ensuring mouse is anesthetized to a surgical plane of anesthesia.
• Quickly transfer mouse to prep cart (or designated prep area) containing appropriate supplies and lay mouse on top of drape.
• Place anesthetic nose cone over mouse's nose to deliver isoflurane (in medical grade oxygen, medical grade air, or room air).
• Wipe dorsal skin of mouse with an alcohol pad and give mouse a subcutaneous injection of cefazolin (33 mg/kg) as a perioperative prophylactic antibiotic.
• Remove mouse from nose cone and place ophthalmic ointment (Neomycin, Polymyxin B Sulfates, Bacitracin Zinc; Akorn Inc.) on each eye.
• Quickly return mouse to the nose cone.
• Shave mouse on its left side from approximately 1 cm beneath xyphoid process all the way down the rear limb circumferentially to the ankle and toward the perineum caudally at its midline (Fig. 3).

**Fig. 4:**
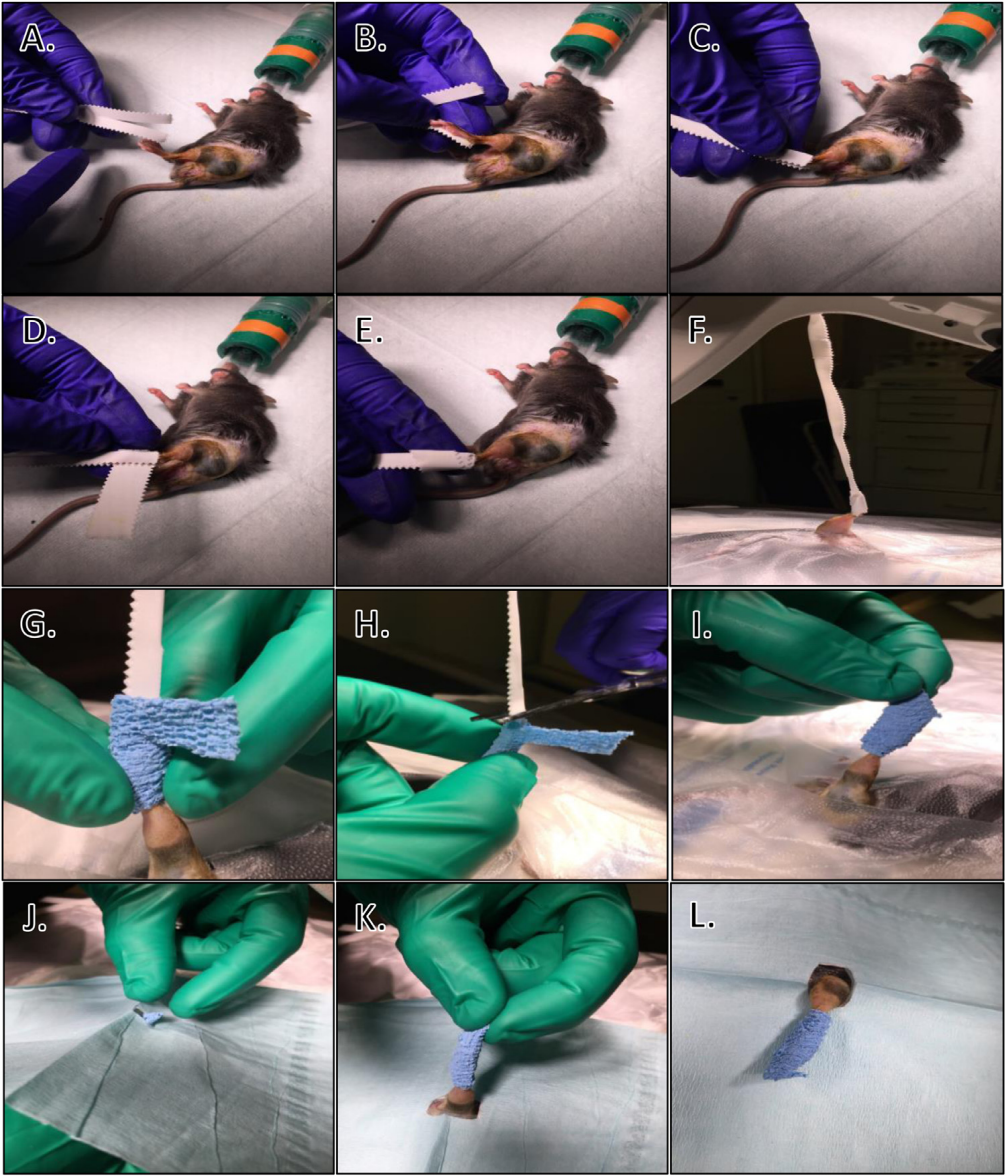
Mouse hanging limb preparation. Stages of hanging limb preparation. A) After the surgical site has been aseptically prepared, begin taping the leg for hanging limb preparation. B) Tape is placed against the foot. C) Tape is closed around the entire length of the foot. D) A second piece of tape is wrapped perpendicularly to the first. E) The leg has been appropriately taped and is ready to be moved to surgical area. F) Mouse is moved to surgical area and laid onto Press'n Seal^Ⓡ^. Additional layers of Press'n Seal^Ⓡ^ are cut and placed overlapping around periphery of prepared surgical site. Limb is hung from operating microscope to allow 360-degree access for surgeon to wrap sterile Vet Wrap around the tape. G) Sterile Vet Wrap is wrapped around the foot by the surgeon, taking care not to touch the non-sterile tape. Note extra length of Vet Wrap to cover open area after tape is cut. H) Tape is cut while surgeon holds limb in place. I) Sterile Vet Wrap has been wrapped successfully around the foot, allowing the surgeon to handle the limb without loss of aseptic technique. J) After the tape is cut, surgeon places sterile drape around the limb. K) Limb is pulled through drape by surgeon. L) Hanging limb preparation is complete and the surgeon has a sterile field and a method to maneuver the limb as required during surgery without breaking the sterile field.
• Open three packages of cotton swabs soaked in Betadine^Ⓡ^ solution (Avrio Health L.P., Stamford, CT) and three alcohol pads and place near mouse so they are ready for use.
• Begin preparing mouse skin: First, add Betadine^Ⓡ^ solution to locations where incision will be placed (one incision medially at stifle and one laterally at mid-femur), then proceed to areas more distal from surgical sites. Next, perform same sterilization technique with alcohol. Repeat these two steps for a total of three times (i.e., Betadine^Ⓡ^, alcohol, Betadine^Ⓡ^, alcohol, Betadine^Ⓡ^, alcohol). *Do all of this while maintaining sterility of mouse's leg by holding mouse's leg up by attaching tape to a stationary object.* Ensure that the skin right up to the edge of the tape is included in the skin preparation procedures.
• Turn off isoflurane delivery to nose cone positioned at the prep area.
• Quickly transfer mouse to surgical table, ensuring leg remains sterile by holding it up by the waterproof first aid tape.
• Turn on isoflurane delivery to nose cone positioned at surgical station and place mouse's nose in the cone.
• Attach the tape on the mouse's leg to the microscope arm so that the mouse's leg is hanging and remains sterile.
• Repeat toe pinch maneuver to ensure that surgical plane of anesthesia has been maintained during transfer.
• Surgeon will then check orientation of mouse to determine if it is correctly positioned for surgery (see Additional Info).
• Prepare one syringe with sterile saline to be used for lavaging surgical site intraoperatively.

Sterile Preparation of Hanging Limb:

• Hanging limb preparation: tear 1” wide waterproof first aid tape in half length-wise using at least 6” of tape. Place end of first strip of tape on ankle on medial side of foot and direct down toward and beyond the toes. Place end of second strip of tape on ankle on lateral side of foot and direct down toward the toes. Press the two pieces of tape together beyond the end of the foot. Take a short piece of tape and wrap circumferentially around the limb from ankle to just beyond the end of the foot to ensure coverage of any haired areas (Fig. 4). Secure the end of the long pieces of tape to a stable, stationary object in order to hold (“hang”) the limb off of the work surface.

Surgical Draping:

• Remove nonsterile gloves, replace PPE, scrub hands or apply a surgical hand disinfectant, and don sterile surgical gloves.
• Cover the mouse in Press'n Seal^Ⓡ^ and cut a hole into a sterile drape. Press'n Seal^Ⓡ^ is very effective at keeping the mouse's nose inside the nose cone and preventing excessive movement of the trunk during limb manipulations.
• Wrap the hanging limb in sterile PetFlex (Andover, Salisbury, MA) from ankle to foot. An assistant carefully cuts the nonsterile tape just beyond the tip of the toes, and the surgeon wraps the sterile PetFlex over the toes to completely cover all of the nonsterile tape. The foot is then directed through the hole in the sterile drape and the drape adjusted to cover the mouse.
• Monitor vital signs and make sure mouse is at a surgical plane of anesthesia.

**Fig. 5:**
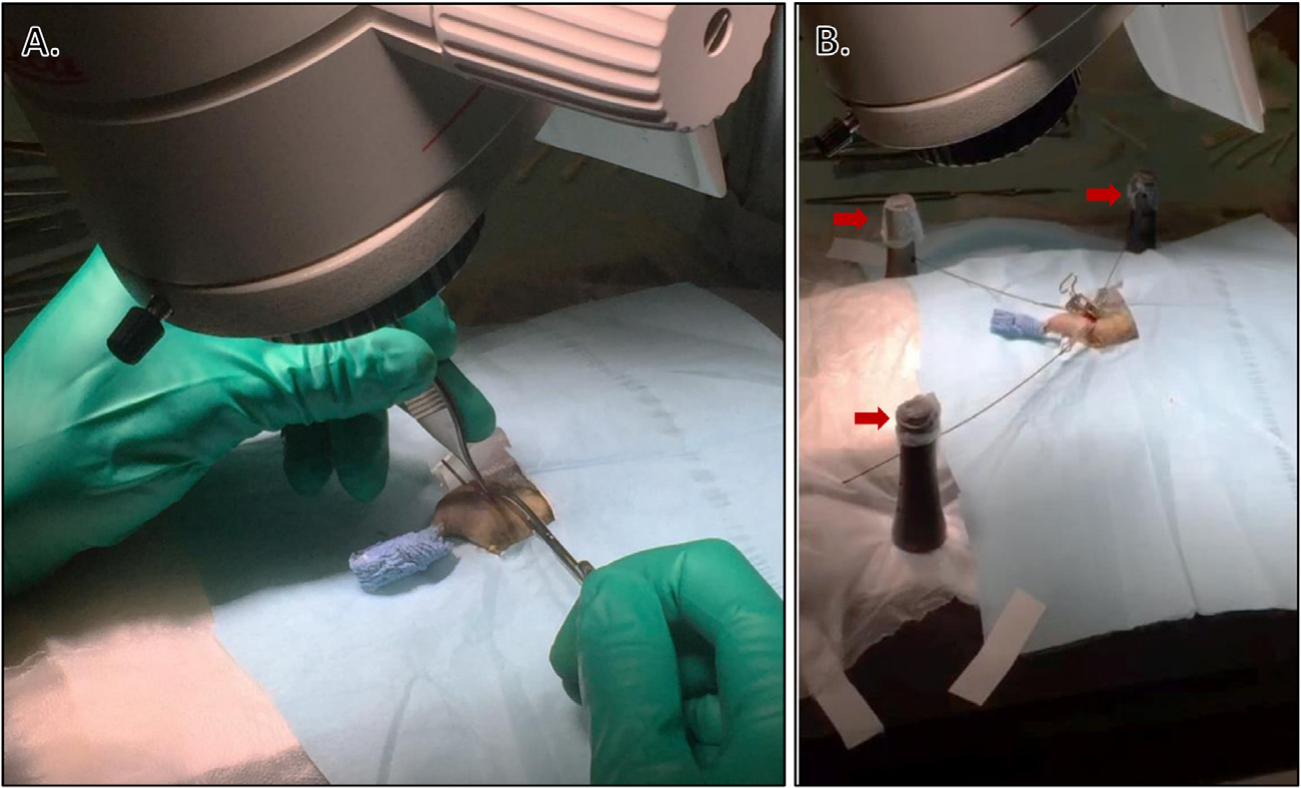
Aseptic surgical field. A) Sterile field consisting of hanging limb preparation, sterile drape, and Press'n Seal^Ⓡ^. B) Magnetic retractors (red arrows) are useful tools for murine surgeries. The retractor arms can be steam sterilized before use in an autoclave.
• The intercondylar fossa of the femur is then engaged using a centering drill bit and the marrow cavity is reamed using a sterile 22G blunted stainless steel needle.
• A 27G stainless steel needle is then used to open the femur proximally for seating of the MouseNail in the marrow cavity.
• The MouseNail is then inserted into the marrow cavity antegrade under continuous manual rotation until the notched distal end of the MouseNail is even with the condyles (the MouseNail extends beyond the notched section for mounting the jig and is eventually sawed off).
• Next, a standard lateral approach to the diaphysis of the femur is performed by making an incision using a scalpel with size 15 blade from the level of the greater trochanter to the patella through skin, subcutaneous tissue, and superficial leaf of the fascia lata along the cranial border of the femur. The biceps femoris muscle is retracted caudally and the vastus lateralis muscle cranially to expose the femur.
• The aiming device (“jig”) of the MouseNail is then positioned onto the MouseNail at the stifle end to allow for insertion of interlocking pins to engage the intramedullary nail.
• Holes for the pins are then drilled using the guide, and, after countersinking, the interlocking pins are inserted (one placed on each side of the planned location of the osteotomy).
• The aiming device is then removed from the MouseNail.
• Osteotomy is created using a sterile Dremel^Ⓡ^ (Dremel, Racine, WI) 1.6 mm bit to generate a fracture gap of 1.6 mm, and surrounding muscle is retracted using magnetic retractors (Fig. 6). The retractor system we use allows autoclaving of the retracting arms.

Surgery:

• Our approach described below was based on modifications to techniques previously described for surgical approaches to the bones and joints in dogs and cats [7].
• Once the mouse is anesthetized and positioned properly, a longitudinal incision using a scalpel with size 15 blade is made along the medial surface of the stifle, and the patella is dislocated laterally to expose the stifle joint (Fig. 5).

**Fig. 6:**
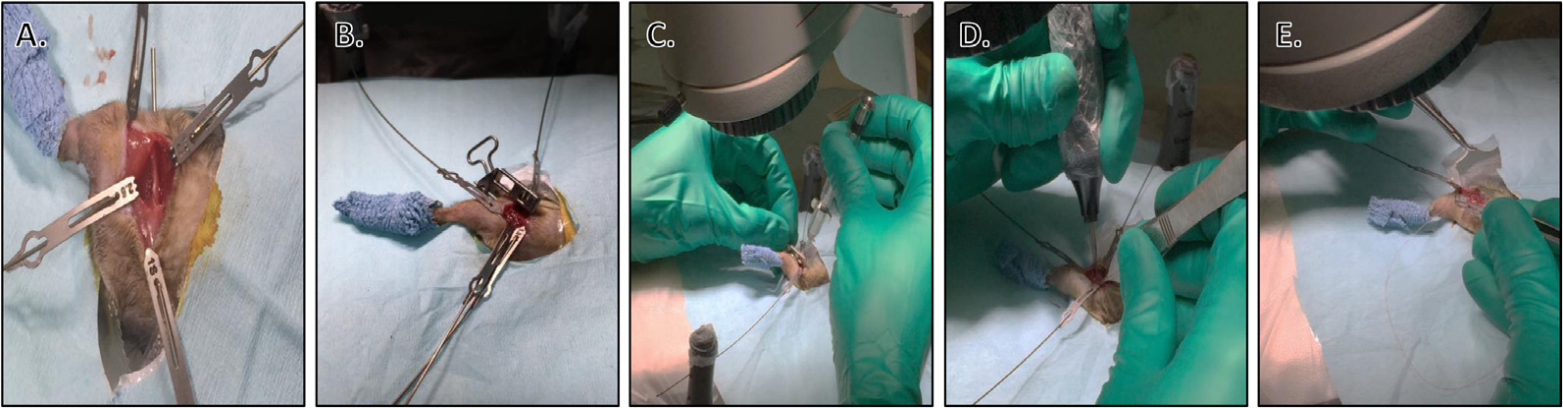
Representative photos of different surgical stages. A) Surgical site with MouseNail installed before placement of aiming device. Magnetic retractors help to keep bone free of surrounding fascia. B) Installed aiming device over MouseNail. C) Countersinking before installation of locking pins. D) Creation of 1.6 mm osteotomy using Dremel^Ⓡ^ drill. E) Suturing of incision site after osteotomy.
• Lavage with sterile saline is performed during this step to irrigate tissues and remove bone micro-debris created during osteotomy.
• The shaft of the MouseNail protruding from the intercondylar fossa and surrounding area is then covered in sterile petroleum jelly, and the shaft is cut using a Dremel^Ⓡ^ mini saw attachment to allow the nail to be flush with the intercondylar fossa (the petroleum jelly is used to catch any metal fines produced during cutting).
• Petroleum jelly and metal fines are lavaged from the surgical site.
• The patellar tendon and patella are then re-positioned in the patellar groove.
• The fascial layers of both incisions are then sutured, followed by skin closure using absorbable sutures.
• Tissumend II synthetic tissue adhesive (Veterinary Products Laboratories, Pensacola, FL) is then applied along the skin incisions.

Recovery:

• Remove Press'n Seal^Ⓡ^ from mouse and place the mouse in a recovery cage.
• Administer analgesic. We use sustained-release buprenorphine (ZooPharm, Laramie, WY) subcutaneously (0.5 mg/kg), which provides 72 hours of analgesia in a single dose.
• Monitor to ensure complete recovery and examine for signs of inflammation, discomfort, or inability to ambulate.
• Continue daily monitoring for at least five days post-operation.
• Mice were examined by microcomputed tomography (micro-CT) at six time points post-surgery (1 day, 7 days, 14 days, 28 days, 56 days, 70 days) to ensure lack of callus formation or bridging of the fracture gap to confirm atrophic nonunion.

## Method validation

**Fig. 7:**
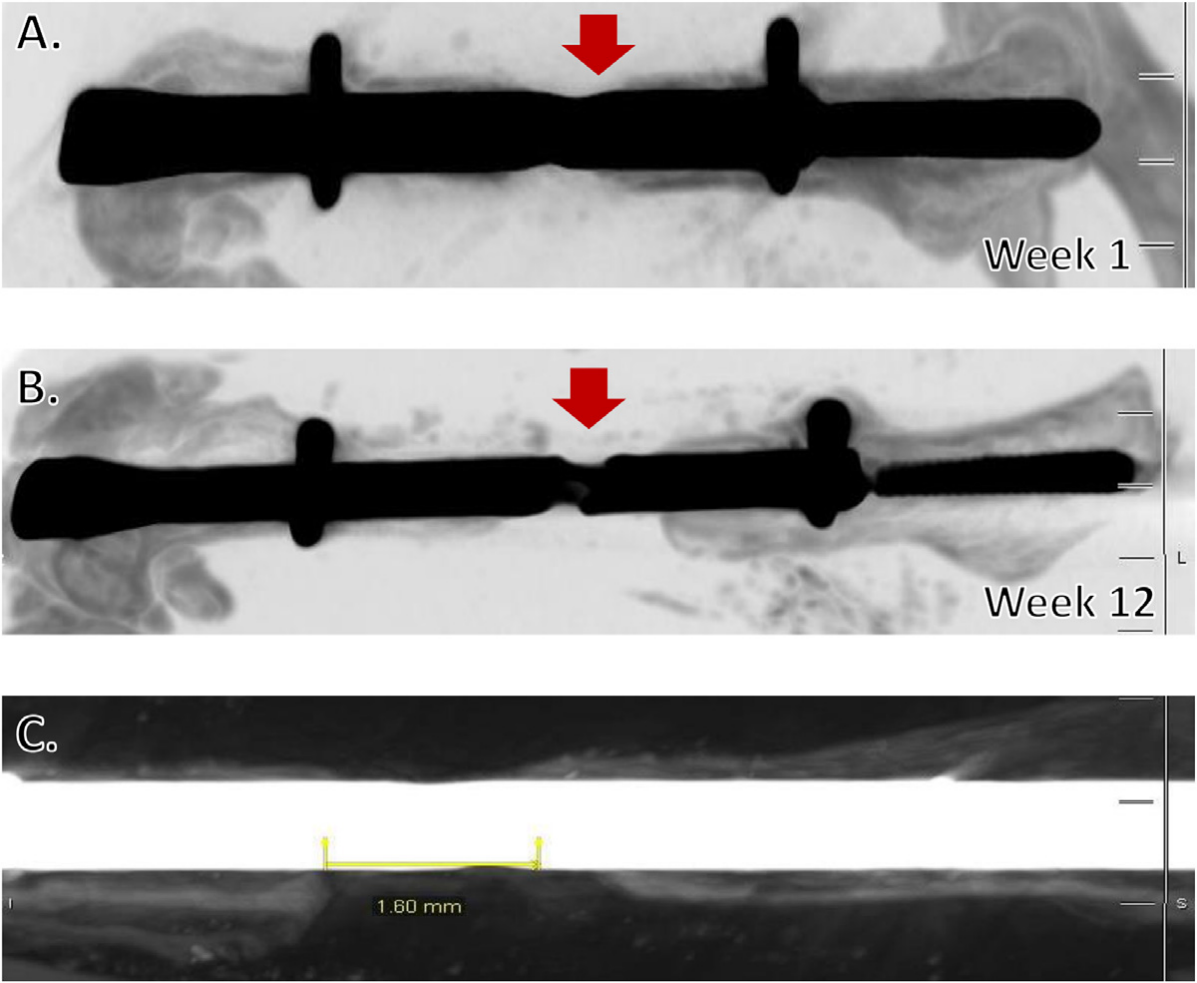
Adapted MouseNail model successfully creates atrophic nonunion. Micro-CT imaging shows that a 1.6 mm fracture gap (red arrow) is maintained one-week post-surgery (A) through twelve weeks post-surgery (B) with little/no callus formation, indicating successful generation of atrophic nonunion (n=4). C) Measurement of fracture gap at twelve weeks post-surgery in mouse with confirmed atrophic nonunion demonstrates that the 1.6 mm gap is maintained.

**Fig. 8:**
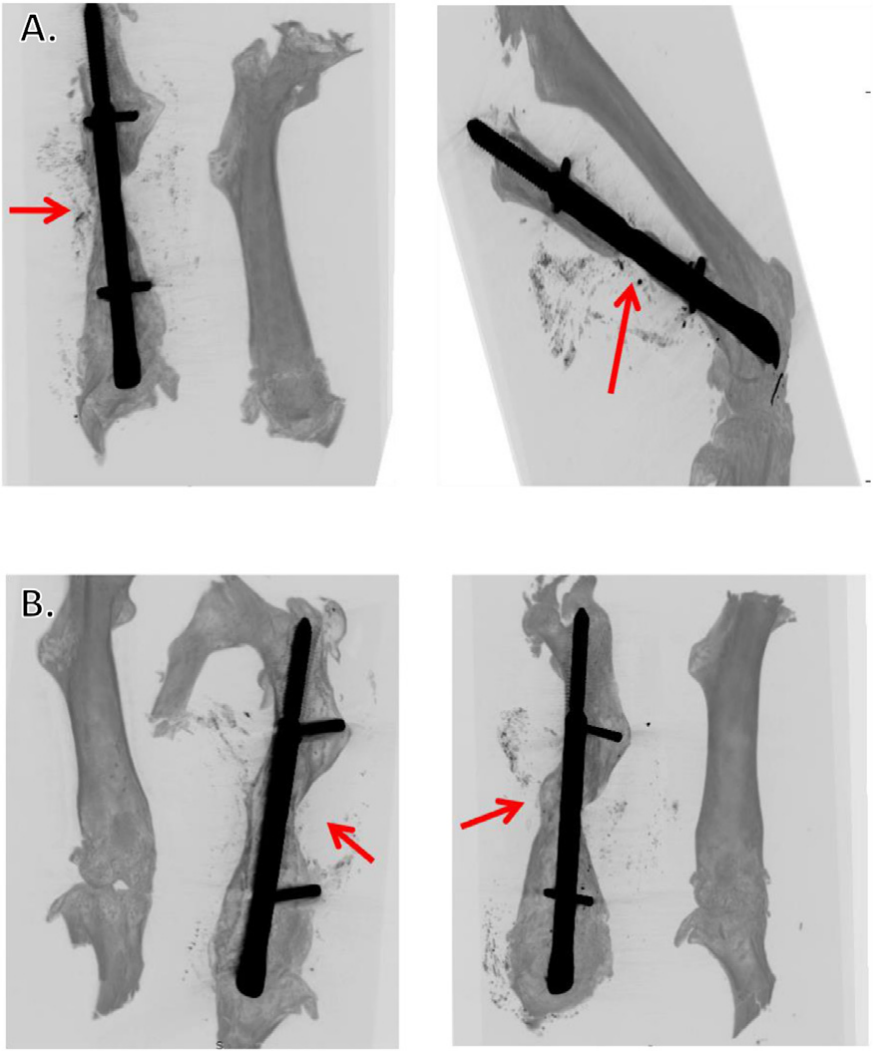
Delayed union is achieved when MouseNail locking pins are not fully installed. A) Mice with locking pins properly positioned. *Ex vivo* micro-CT images at twelve weeks post-surgery confirm atrophic nonunion with no/minimal fracture bridging (arrows). Contralateral limbs are shown beside. B) Mice with improper positioning of locking pins. *Ex vivo* high-resolution micro-CT images at twelve weeks post-surgery demonstrate bony union (arrows) with bony outgrowth surrounding the improperly positioned locking pin. Contralateral limbs are shown beside.

**Fig. 9:**
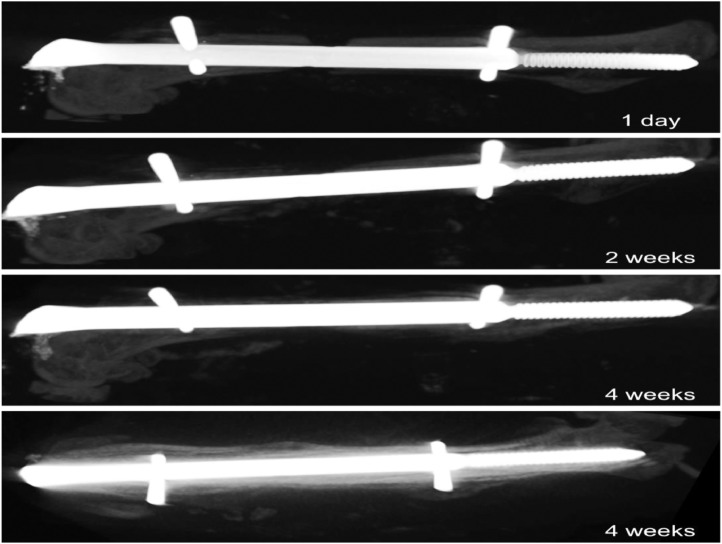
Normal fracture healing is achieved when 0.8 mm fracture gap is generated. Micro-CT images show clear fracture gap at one day and two weeks post-operation. However, by four weeks, the fracture gap has healed and bony union is observed, suggesting 0.8 mm fracture gap results in standard healing of murine femoral fracture. Top-down view at 4 weeks is added for clarity.

Methods developed to establish a sterile surgical field, including hanging limb preparation and use of Press'n Seal^Ⓡ^, resulted in all animals surviving the operation without any evidence of early complications due to bone or soft tissue infection. Our methods also met expectations of the IACUC and regulatory standards. The miniaturization and application of principles for a hanging leg preparation allowed the limb to be manipulated circumferentially throughout the surgery, facilitating incisions on opposite sides of the limb and increasing precision of entry into the medullary cavity, without compromising sterility. Together, these refinements led to a standardized, cost-effective method for establishing an aseptic surgical environment for murine orthopedic studies. Using the MouseNail kit and novel modifications, we have successfully created an aseptic femoral atrophic nonunion mouse model based on 1.6 mm critical-sized defect. Micro-CT analysis of fracture gap demonstrated absence of callus formation and minimal gap bridging in all animals past the time of normal healing (four weeks) and up to twelve weeks post-operation (Fig. 7). These results indicate that a fracture gap size of 1.6 mm results in successful generation of atrophic nonunion (Fig. 7C; n>20). We have observed that both locking pins need to be fully installed for atrophic nonunion formation; otherwise, a delayed union forms, which is likely due to biomechanical instability (Fig. 8). We have also tested a smaller fracture gap size of 0.8 mm, but this resulted in standard murine femoral fracture healing, with mice achieving union as quickly as four weeks post-operation (n=3) (Fig. 9). The adaptation of preclinical nonunion studies to the mouse model allows for more elegant mechanistic studies through the use of transgenic and genetic animal models not available in larger species. The novel modifications developed in this study can be applied to increase reproducibility of this technically challenging surgery, understand the basic mechanisms of nonunion repair, and utilize this knowledge to examine the efficacy of clinically relevant therapeutics. For example, our research group is interested in using the novel model developed herein to examine the ability of stem cells to prevent atrophic nonunion formation.

## Additional information

We have provided additional background material to aid researchers interested in performing murine orthopedic survival surgery. We have described unique features of murine long bone anatomy and provided detailed pre-operative through post-operative recommendations and considerations.

### Unique features of murine long bone anatomy

**Fig. 10:**
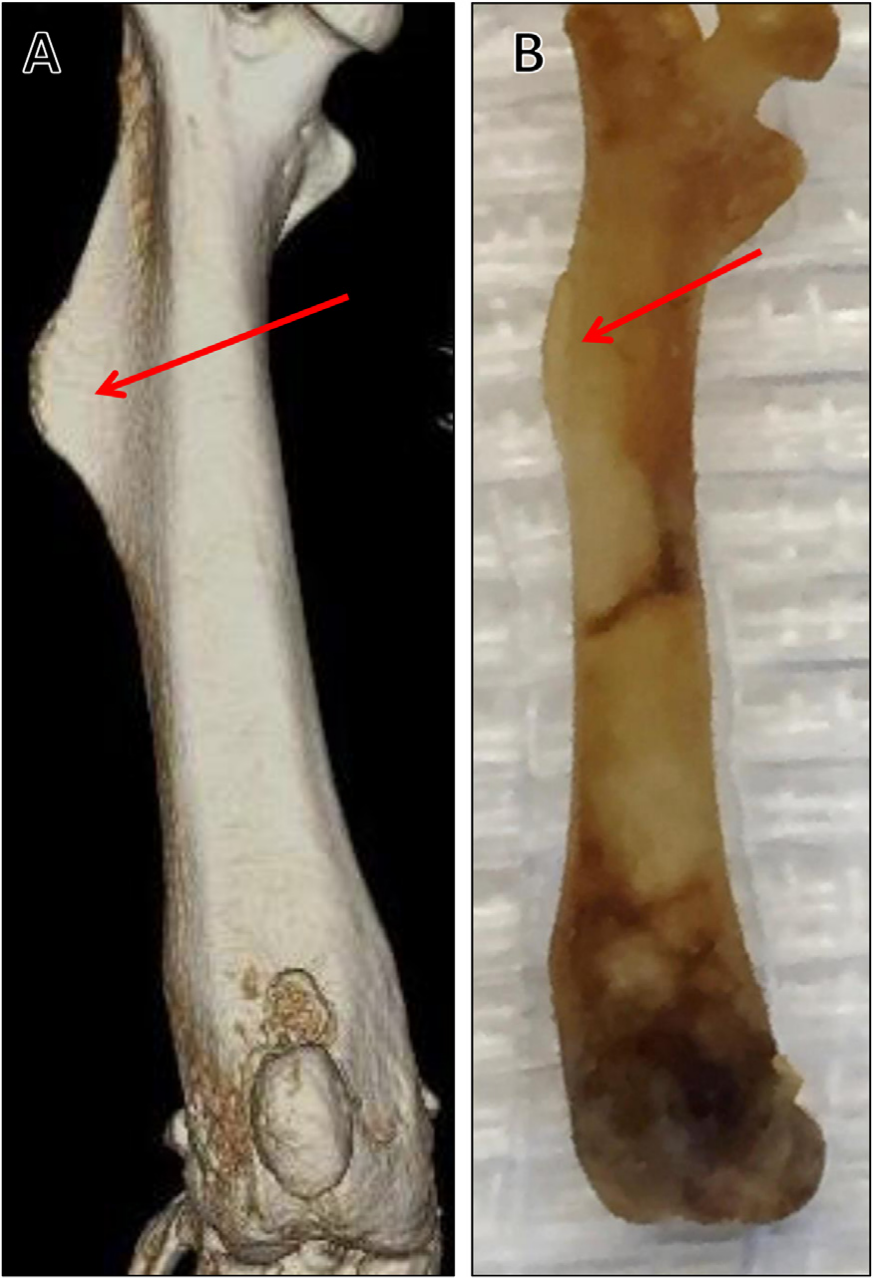
Third trochanter of mouse femur. A) Micro-computed tomography image demonstrating large third trochanter that appears as a flaring of the diaphysis (red arrow). This anatomic feature may cause difficulty to the surgeon when placing pins through the proximolateral diaphysis. B) A dermestid beetle-cleaned mouse femur. These specimens allow trainees to familiarize themselves with distinct nuances of rodent anatomy, such as the third trochanter (red arrow).

**Fig. 11:**
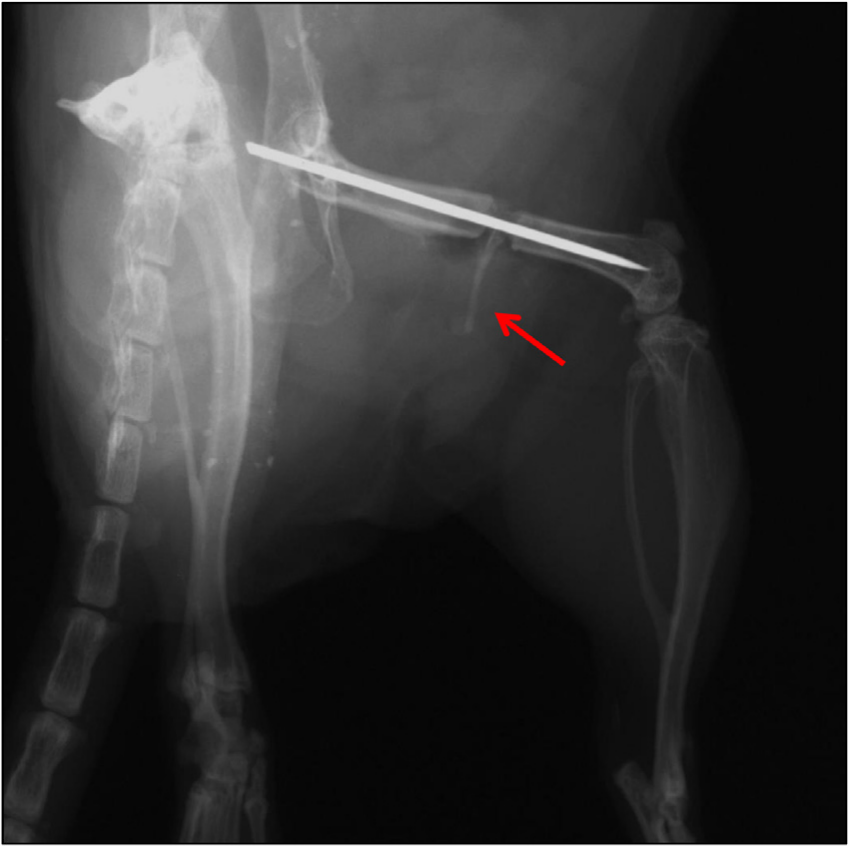
Mouse os penis. Radiographic image demonstrating the os penis in a male mouse (red arrow). The mouse must be positioned to prevent the os penis overlapping the fracture gap during imaging.

There are unique features of mouse long bones that both novice and experienced surgeons must be aware of before performing orthopedic surgical procedures. The mechanical properties of bone are determined, in part, by bone geometry, bone mineral density, and structure. Thus, understanding these anatomical differences is important during surgery and for post-operative analyses. For example, mice lack Haversian systems. Instead, they have resorption cavities for bone remodeling and have persistent growth plates throughout their life span. In murine fracture studies, the femur is most commonly used, due to the tibia being smaller, more difficult to stabilize, triangular in shape, and possessing a declining diameter distally and bowed longitudinal axis. By comparison, the mouse femur is a tubular bone with a relatively consistent diameter. Different fracture sites along the femoral diaphysis result in similar callus and healing responses, mitigating fracture standardization issues associated with the tibia [8]. The mouse proximal femur differs from other animal species by the size, shape, length, and width of the femoral neck, as well as the angle between the femoral neck and diaphysis [9]. Compared to companion species, such as dogs and cats, the mouse has a large, angular third trochanter on the proximolateral femur, presenting a “flaring” of the diaphysis, as opposed to a flat surface (Fig. 10). This feature can cause technical complications during surgery, particularly when pins or plates need to be applied for fracture stabilization. In mice, the fibula fuses to the tibia distal to midshaft, whereas, in humans, it extends distally to connect near the ankle [10]. In addition, three-dimensional musculoskeletal geometry and architecture of the mouse hind limb has been performed using I_2_KI-enhanced micro-CT. This demonstrated a proximo-distal gradient of muscle architecture within the hind limb, which is likely an adaptation to improve locomotor efficiency and motor coordination [11]. One potential confounding anatomical landmark in mice is that males, after puberty, have an os penis that is comprised of hyaline growth cartilage proximally and bone distally. These skeletal elements likely provide necessary rigidity required for murine mating. However, as this is mineralized tissue, it will be clearly visible during x-ray or micro-CT analyses and, thus, may affect visibility of fracture sites (Fig. 11) [12]. These are examples of some of the intricacies associated with murine skeletal anatomy that the surgeon needs to be aware of, as they may cause technical complications within the orthopedic procedures and affect outcomes, particularly when attempting to model specific human conditions.

### Pre-operative considerations

There are a number of pre-operative decisions and actions that must be taken before surgeries can commence. These include: properly preparing a dedicated surgical area, sterilizing surgical instruments, training a surgical assistant, and arranging appropriate materials within the different surgical support areas.

#### Dedicated surgical area

**Fig. 12:**
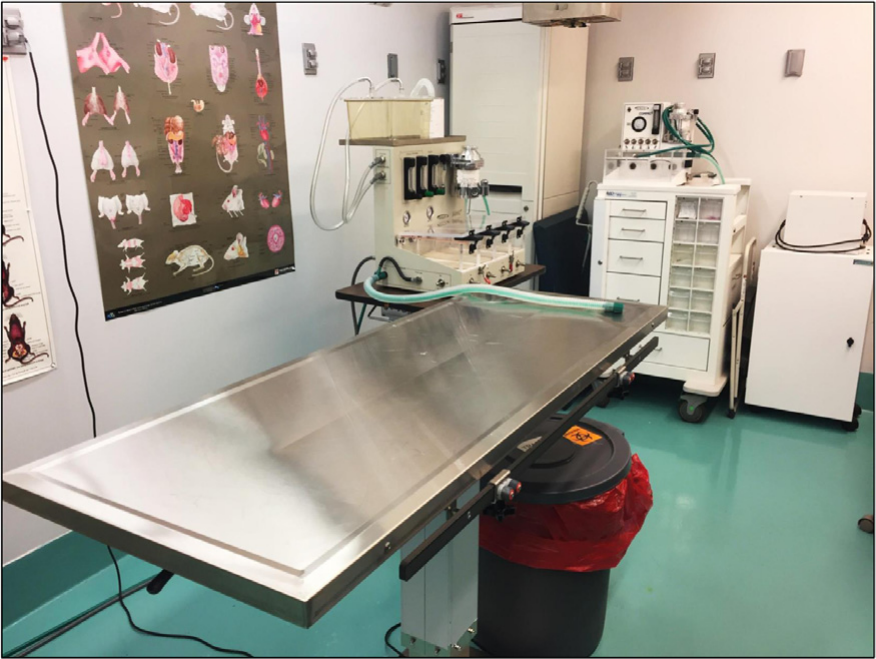
Centralized, dedicated rodent surgical suite. Example of a dedicated rodent surgical suite.

Although recommended, rodent survival surgery does not require a dedicated surgical suite like the one we use at our facility (Fig. 12). However, surgeries should be performed in a designated area of the laboratory (preferably a separate room) away from normal traffic flow, windows, and other work areas that may experience turbulent airflow. At the time of surgery, this area should be well-lit, organized, and free of non-relevant instruments and materials. At a minimum, the bench top should be cleaned thoroughly with a disinfectant, such as ammonium chloride-, chlorine-, or peroxide-based disinfectants, before the start of any procedure. Alternatively, a high-efficiency particulate air (HEPA)-filtered hood can be used for the surgical area, as long as the airflow does not desiccate exposed tissues.

#### Preparation of surgical instruments

Surgical instruments, particularly those used for rodent surgery, may have small or delicate tips. Thus, autoclavable tip guards should be utilized to protect instruments. Instruments with damaged tips or dull blades should be immediately discarded and replaced, as their use may increase surgical trauma. In order to sterilize surgical instruments, a number of techniques can be employed [13]. These include use of a steam-based autoclave, dry heating, chemical sterilization, ethylene oxide gas, or radiation. Autoclaving is the gold standard for sterilization but only works on instruments that can be heated to extreme temperatures. Instruments should be wrapped and autoclaved before the first surgery of the day. Some chemical sterilants may be corrosive to metal and tissues, thus should be properly rinsed or aerated after sterilization per product manufacturer instructions. Common laboratory disinfectants, including ethyl or isopropyl alcohols, are not sterilants, as they lack sporicidal activity, cannot penetrate protein-rich materials, and lack ability to kill hydrophilic viruses [14]. Glass bead sterilizers are commonly used to sterilize the tips of previously autoclaved surgical instruments between animals on the same day for serial surgeries. However, instruments must be allowed to cool after being placed inside of the hot bead sterilizer to avoid thermal injury to tissues. No matter the method used, the primary goal of sterilization is to remove all traces of microbial life from the surgical instruments. Methods chosen for use should be consistent with local IACUC policy.

#### Surgical assistant

A surgical assistant is extremely helpful for maintaining asepsis during any complex orthopedic surgical procedure. The assistant can prepare the animal, control anesthesia, administer medications, monitor recovery, and maintain records. This allows the surgeon to remain focused on their tasks, limits the risk of breaking sterility in the surgical field, and optimizes and increases work flow, leading to more surgeries being performed each session and reducing risk of failure of asepsis. However, if having a surgical assistant is not possible, then the surgeon should take care to sterilize or prepare surfaces and instruments that may have to be touched outside the surgical field. For example, the knob on the anesthesia machine can be covered in autoclaved aluminum foil or Glad^Ⓡ^ Press'n Seal^Ⓡ^ to prevent cross-contamination. It is worth noting that our hanging limb preparation, as described, requires a surgical assistant in order to not compromise sterility, so, if a surgical assistant is not available, then modifications to our proposed method would have to be made.

#### Arrangement of surgical area and necessary supplies

The surgical area should be arranged into three distinct zones: preparation, surgery, and recovery. Within the preparation area, supplies should be cleaned and assembled for use.

#### Animal preparation area

**Fig. 13:**
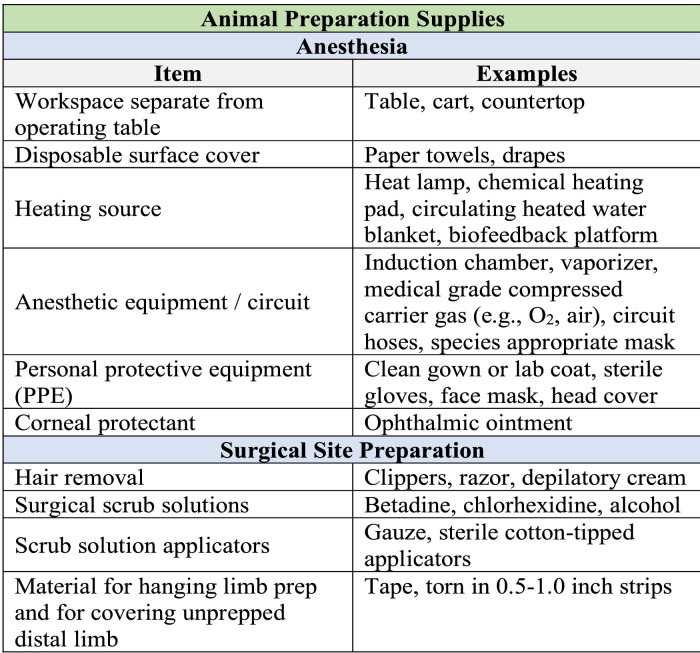
Supplies required for surgical preparation of animal. A standardized surgical supply list is valuable during preparation for animal surgery.

The animal preparation area should be physically separate from the surgical area but is often located in the same room. Recommended general supplies are outlined in Fig. 13.

#### Pre-operative routine

Rodents do not typically need to be fasted before surgery, as they are incapable of vomiting. In addition, due to their high metabolic rates, fasting may rapidly deplete energy reserves, thereby exacerbating hypothermia. A pre-surgical evaluation should be performed to omit any animals that appear to have a pre-existing condition. As bone structure and biomechanical characteristics change with age, strain, and sex, it is recommended to use strain-, sex-, and age-matched mice within the same study.

Preemptive analgesia should be administered to the mouse using an appropriate dose based on body weight. It is important to note that mice have a high surface area to body volume ratio and rapid metabolism. As such, pharmacological doses of analgesics typically need to be delivered at higher doses on a per kilogram basis than in larger species. Similarly, dehydration can occur much more rapidly. Although there are a number of analgesics that may be used, we recommend using buprenorphine pre-operatively for murine orthopedic procedures. In our laboratory, buprenorphine is preferred over another commonly used class of analgesics, non-steroidal anti-inflammatory drugs (NSAIDs), because NSAIDs have been shown to have deleterious effects on fracture healing and have been clinically shown to increase the rate of delayed union and nonunion in fracture patients [15], [16], [17], [18], [19].

Perioperative antibiotics should be administered only if sound rationale for their use is employed. In a research setting where orthopedic surgery is usually performed on young, healthy subjects, reasons for needing perioperative antibiotics include clean surgical procedures in which the incision is open longer than 90 minutes, a permanent implant is placed, or the potential consequences of infection would be catastrophic (e.g., central nervous system or joints). Most surgical site infections are thought to result from contamination from skin bacteria of the patient. In mice, the most common skin bacterium is *Staphylococcus intermedius* [20]. Therefore, an appropriate perioperative antibiotic choice is a first-generation cephalosporin, such as cefazolin. Cefazolin is an effective prophylactic antibiotic, with high serum levels, long duration, efficient bone penetration, and robust coverage for gram-positive bacteria, like *Staphylococcus* sp., and limited gram-negative efficacy, while remaining at a reasonable cost [21,22]. In our facility, we typically administer cefazolin to rodents subcutaneously 30-40 minutes before incision and repeat the dose every 90 minutes, if needed, until the incision is closed.

Next, the mouse should be anesthetized, which can be performed using either inhalant or injectable anesthetics. Inhalant anesthetics have the advantages of ease in maintaining anesthetic depth, acceptable cardiopulmonary stability, and minimal metabolism, biotransformation, and excretion. Drawbacks include hypotension, the need for specific anesthetic equipment and mitigation of risk of personnel exposure to waste anesthetic gas, and cost. The most common inhalant anesthetic is isoflurane. Ensure your isoflurane machine is full prior to the day's surgeries and that there is a method in place for scavenging of waste anesthetic gases (WAG), such as a WAG snorkel, other active scavenging to the outdoors, or passive charcoal canister. If you are using injectable anesthetics, a mixture of ketamine and xylazine or dexmedetomidine are commonly used and provide a low level of analgesia into the postoperative period. Although intraperitoneal injection is standard, subcutaneous injection may be a safer approach that diminishes pain, peritoneal irritation, and ameliorates risk of abdominal organ perforation [23]. Each animal should be weighed and dosed according to its body weight if using injectable anesthetics. Injectable anesthetics are inexpensive, easy to use, mitigate exposure-related health effects to surgical personnel, and make animal manipulation easier [24] but may require multiple doses, increasing risk of hypothermia, prolonged metabolism, and recovery. In general, you should consult your facility's veterinarian to determine the best anesthetic for your protocol. Once an anesthetic has been given, mice should be monitored to ensure they are in the appropriate anesthetic plane, which can be assessed by toe, ear, or tail pinch, as well as respiratory rate, depth, and rhythm. If the animal reacts to pinching, then it has not been adequately anesthetized.

After the mouse has been anesthetized, sterile ophthalmic ointment should be applied to both eyes to prevent corneal drying and protect corneas from stray fur during clipping. Once these tasks have been performed, preparation of the incision site can begin. A rule of thumb is to shave an area three times the size of the incision site. Extreme care must be taken to remove all fur cleanly from the skin surface in the area of the surgical site, and the underlying skin should be meticulously decontaminated. Clipping is a common method for removing fur, but, often, the first pass of the clipper leaves hair stubble that must be clipped again or at a different angle. For small surgical sites, plucking may be advantageous because it removes the entire hair, including root, from the follicle. For very large sites, depilatory cream may be required or used after initial clipping. After clipping, rubbing with a sterile alcohol pad or the use of adhesive tape can remove any remaining loose hair. Decontamination of the incision site can then be performed on the mouse, with a general approach being three alternating applications of 70% alcohol and either povidone-iodine (i.e., Betadine®) or chlorhexidine, typically using sterile cotton swabs. Begin at the center of the incision site and progress outward. Care should also be taken to avoid excessive wetting of the skin and non-surgical areas of the animal, as this can induce hypothermia. Importantly, isopropyl alcohol has been found to equilibrate mouse body temperature faster than use of room-temperature saline during surgical site preparation [25]. This effect is likely due to isopropyl alcohol not leaving any liquid residue; thus, after evaporation, it no longer influences temperature measurements. However, unlike with saline, care must be taken to avoid oversaturation with isopropyl alcohol because it can be rapidly absorbed through the skin and may result in overdosing [26].

If there is a requirement of the surgery, as is the case in our atrophic nonunion femoral fracture protocol, to access both the medial and lateral aspects of the limb while maintaining sterility, a hanging limb preparation can be performed after the surgical site has been properly prepared (Figs. 3 & 4). The mouse can then be moved to the surgical area by the surgical assistant and covered with a sterile surgical drape by the surgeon. The assistant can tape the edges of the drape away from the surgical field, such as the underside of the operating table, if necessary.

### Surgical operation

#### Surgical Area

Within the sterile surgical area, supplies should be assembled and organized. A list of required general surgical supplies has been provided (Fig. 1).

#### Operative routine

For the surgeon, clean personal protective equipment should be donned, which includes a clean lab coat or surgeon's gown and surgical mask, while long hair should be tied back or covered. Surgical instruments can be unwrapped, with care taken to avoid touching the inside of the packaging, as this risks contamination, and dropped onto a sterile drape on the operating table. The surgeon can then perform a surgical scrub of their hands and put on sterile surgical gloves without touching the exterior of the gloves. This can take some practice to perform correctly. If sterile surgical gloves are not an option due to cost or availability, then nitrile exam gloves cleaned with 1% hydrogen peroxide and 0.08% peracetic acid were shown to be 94% negative for bacterial contamination, while 98% of nitrile exam gloves that were autoclaved met performance expectations, which were similar rates to those observed for sterile surgical gloves [31]. Thus, either sterile surgical gloves or exam gloves treated to meet acceptable asepsis are permissible for rodent surgery, depending on institutional rules.

The mouse should be positioned appropriately. The surgical site should be centered within the field, particularly if a surgical microscope is needed. The limbs should not be excessively stretched or tensed, as this may traumatize joints, stretch nerves, and/or impair breathing. Importantly, if using an operating microscope, the surgeon should ensure the entire required field of view is within focus pre-operatively, as it can be difficult to move the mouse after it has been draped and covered without compromising sterility. Once the mouse is positioned and appropriately draped, the surgeon may begin the procedure. Sterile surgical drapes are used to cover part of or the entire rodent. The drape protects against accidental contamination by establishing a clearly visible sterile field. If the laboratory has sterile drapes manufactured for larger animals, these can simply be trimmed to size for rodents. In addition, Glad^Ⓡ^ Press'n Seal^Ⓡ^ provides a sterile, inexpensive, and effective alternative to traditional surgical drapes to cover the surgical field and mouse [6]. The sticky side is placed toward the mouse. The advantage to Press'n Seal^Ⓡ^ is that it is clear, thereby allowing for easy monitoring, and can be wrapped around objects, such as microscope knobs, instruments, or nonsterilizable equipment. It is also useful for holding the nose of the mouse inside the nose cone, even when the body and rear legs are extensively manipulated. Further, Press'n Seal^Ⓡ^ is sterile upon opening. We use Press'n Seal^Ⓡ^ extensively in our atrophic nonunion femoral fracture surgeries due to its ease of use.

Mice lose body heat rapidly after shaving and due to limited fat storage and energy reserves, so hypothermia is a frequent complication during surgery. A circulating water heating pad, air-warming blanket, feedback warming platform, or heat lamp should be used to help maintain body temperature, particularly for longer surgeries. Care must be taken, particularly when using heating lamps or heating packs, to avoid excessive localized heat, which can result in thermal injury. We place a heating pack (Cooper Surgical) under the mouse and cover the pack with Press'n Seal^Ⓡ^.

#### Recovery area

**Fig. 14:**
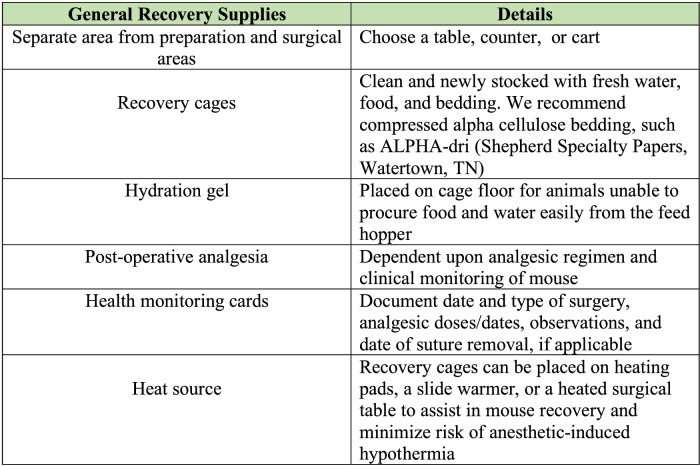
Common supplies required post-operatively. Supplies required post-operatively are outlined with pertinent details.

**Fig. 15:**
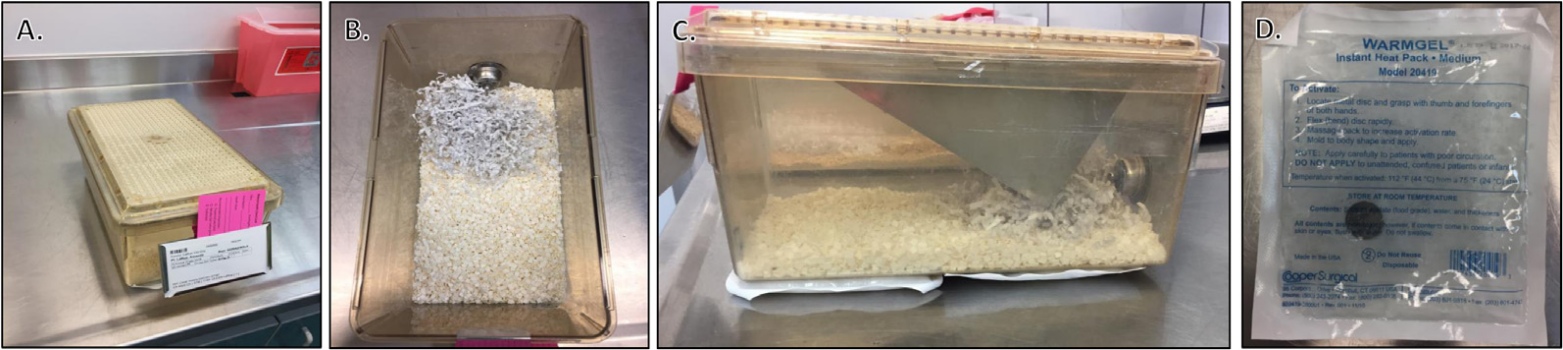
Preparation of post-operative recovery cage and area. A) Ensure the recovery area is on a separate work table from the surgical area. Each mouse undergoing surgical procedure should be singly housed in a clean cage with fresh food and water. B) ALPHA-dri^Ⓡ^ bedding is preferred for mice post-operation. It is more comfortable and allows for easy identification of any exudate from surgical site that may indicate infection or wound opening. C) During recovery, heating pads may be placed under the cage to prevent hypothermia. D) An example of an instant heat pack that can be used.

We have outlined the general recovery supplies that are commonly required (Fig. 14). Within the recovery area, supplies should be assembled and organized. The post-operative recovery cages should be clean, with ample bedding, food, and water (Fig. 15). For surgeries expected to be moderately or severely painful, placing food and/or hydration gel on the cage floor may be beneficial. We have found ALPHA-dri^Ⓡ^ bedding to be the best bedding to use for mice after orthopedic surgery, as it provides comfort for the mouse, good absorbency, and easy observation of any wound discharge from the mouse against its white color. It can be useful to ensure each station is fully equipped and prepared for all surgeries that will be performed that day, thereby allowing the surgeon to continue uninterrupted between surgeries.

### Operative considerations

Position the shaved area of the mouse limb within the center of the surgical field. Anesthetic depth should be assessed before the first incision and routinely during the procedure by lack of response to toe pinch, lack of palpebral reflex, and rhythmic deep respirations. Monitor vital signs throughout the procedure. Thermal support is recommended to limit risk of hypothermia.

Crystalloid fluids given to replenish blood or fluid losses during surgery should be sterile and warmed prior to administration. If a surgical assistant is available, they can be responsible for fluid preparation and administration.

Care should be taken to ensure gentle tissue handling, minimal dissection, and effective hemostasis, as doing all of these will limit risk of surgical complications. Self-retaining magnetic retractors are beneficial for small surgical fields, as compared to having a surgical assistant handle retractors. The retractor arms should be sterilized pre-operatively.

Deep tissue layers should be closed using absorbable sutures in a continuous or interrupted pattern. Mouse skin wounds can be closed with a buried suture pattern followed by skin glue to ensure incisions remain sealed. If external skin suture is used, nonabsorbable monofilament suture should be used to close the skin wound to prevent environmental bacterial pathogens from wicking on braided suture into the incision site. Mice also tend to chew external sutures. Therefore, we prefer a subcuticular closure reinforced with skin glue. Stainless steel wound clips may also be used. Maintain sterile suture material within the surgical field at all times.

When choosing suture size, a 4-0 suture thickness or smaller is generally properly sized for mice. Cutting and reverse cutting needles are generally reserved for subcuticular or skin suturing. Non-cutting, round, or tapered needles are appropriate for suturing tissues deep to the skin that can be easily torn. If tissues deep to the skin need to be sutured, then an absorbable material should be used.

### Post-operative considerations

**Fig. 16:**
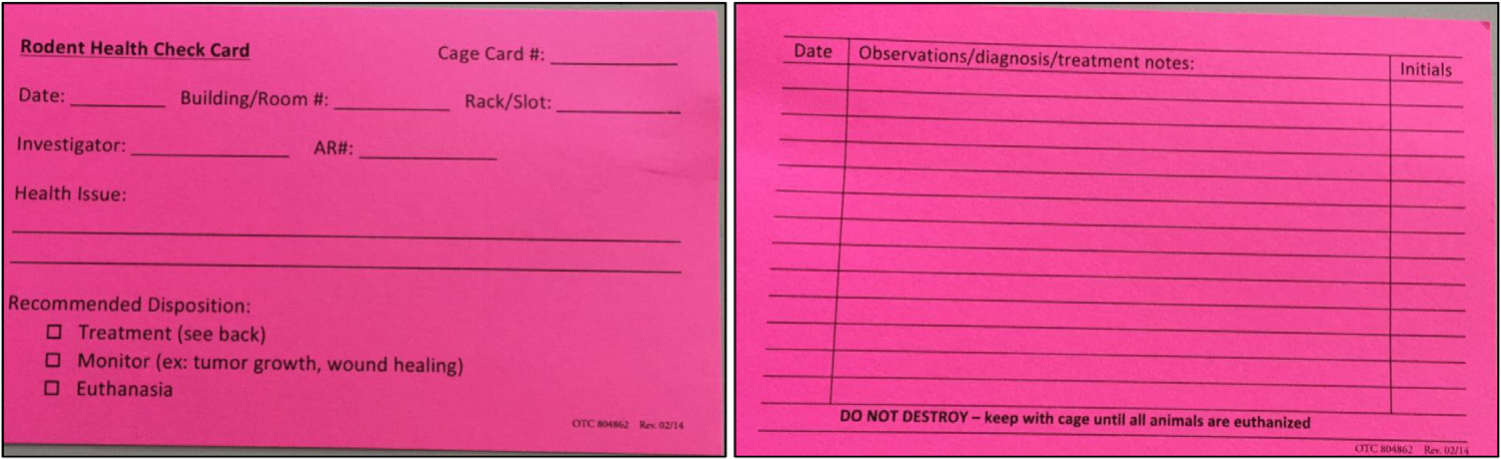
Example of rodent health check card for post-operative monitoring. Rodent health check card should be placed behind cage identification card. This card easily identifies mice that have undergone a procedure to the animal facility staff and allows for clinical monitoring over time.

After surgery, ensure animals recover appropriately from anesthesia and do not become hypothermic. Placing a heating pad under half of the cage during initial recovery can be beneficial. Analgesia should be maintained for 48-72 hours post-operation for most orthopedic procedures. Additional analgesics may need to be given if animals show signs of pain or distress, including decreased food and water consumption, weight loss, aggressive behavior, vocalization, unkempt appearance, trembling, or exhibition of porphyrin staining. Sustained-release buprenorphine may be used post-operatively for analgesia. This drug slowly releases for up to 72 hours after delivery and provides blood levels greater than 1 nanogram/milliliter in rodents, thus limiting the need for multiple doses and handling of mice after orthopedic surgery, which is optimal for limiting manipulation-related postoperative pain [27], [28], [29]. Depending on the extent of surgical trauma, food and hydration gel may need to be placed on the cage floor. Animals should be monitored at least once daily for a minimum of five days post-operation, and frequency may be adjusted depending on the surgery. The animals should be monitored for signs of pain and distress, and the incision site should be checked for incision integrity, including intact sutures and closed wounds, redness/swelling, incisional discharge, or pain at the incision site (Fig. 16). Hydration can be monitored by tenting the dorsal skin of the mouse. If properly hydrated, the skin should quickly fall back into place when released.

### Post-operative monitoring

Adequate surgical records should be maintained for each animal or group of animals treated identically. Descriptions of animals, the type of surgery performed, any treatments, a description of any complications (e.g., infection, distress, pain) that arose during the entirety of the surgical procedure, date when skin sutures or wound clips are removed, and date when animal is able to ambulate should all be documented, as these may become important as data is generated and analyzed. We prefer singly housing mice immediately after surgery, both to avoid trauma caused by cage mates and to facilitate monitoring of each mouse.

If, at the end of surgery, one can answer yes to each of these questions, then the surgery was a success:

1) Was the model properly created?
2) Did the mouse experience pain or distress at levels less than or equal to those expected?
3) Were there no signs of infection or surgical complications?

As one final recommendation, we have found that using a mesh handling device to move mice post-operation is a useful approach to minimize pain, distress, and trauma. We typically use this device to move mice from cage to induction chamber when performing micro-CT scans [30].
